# The Impact of Direct-Acting Antiviral Therapy on the Risk of Recurrence after Curative Resection in Patients with Hepatitis-C-Virus-Related Early Stage Hepatocellular Carcinoma

**DOI:** 10.3390/medicina58020259

**Published:** 2022-02-09

**Authors:** Yu-Syuan Chen, Kuo-Hsuan Huang, Pei-Ming Wang, Ching-Hui Chuang, Chee-Chien Yong, Yueh-Wei Liu, Pao-Yuan Huang, Chih-Chien Yao, Yen-Po Lin, Ming-Chao Tsai

**Affiliations:** 1 School of Medicine, Chung Shan Medical University, Taichung 40201, Taiwan; dante44442@gmail.com (Y.-S.C.); youcryforlol@gmail.com (Y.-P.L.); 2 Division of Hepato-Gastroenterology, Department of Internal Medicine, Kaohsiung Chang Gung Memorial Hospital, Chang Gung University College of Medicine, Kaohsiung 83301, Taiwan; ken10020@hotmail.com (K.-H.H.); paoyuan813@gmail.com (P.-Y.H.); chihchienyao@gmail.com (C.-C.Y.); 3 Department of Family Medicine, Kaohsiung Chang Gung Memorial Hospital, Kaohsiung 83301, Taiwan; wangpeming@yahoo.com.tw; 4 Department of Nursing, Meiho University, Pingtung 91202, Taiwan; helen.ch.chuang@gmail.com; 5 Liver Transplantation Center and Department of Surgery, Kaohsiung Chang Gung Memorial Hospital, Chang Gung University College of Medicine, Kaohsiung 83301, Taiwan; yong3980@hotmail.com (C.-C.Y.); anthony0612@me.com (Y.-W.L.); 6 Graduate Institute of Clinical Medical Sciences, College of Medicine, Chang Gung University, Taoyuan 83301, Taiwan

**Keywords:** DAA, hepatocellular carcinoma, recurrence

## Abstract

*Background and Objectives*: The impact of direct-acting antiviral (DAA)-based regimens on the recurrence of hepatocellular carcinoma (HCC) after successful curative hepatectomy is controversial. Aims: This study aimed to assess the association between DAAs treatment and recurrence risk in HCC after resection. *Materials and Methods*: We retrospectively assessed 152 cases of early stage (BCLC stage 0/A) hepatitis C virus (HCV)-related HCC (HCV-HCC) that underwent resection with curative intent between 2001 and 2019 at Kaohsiung Chang Gung Memorial Hospital; 48 cases achieved a sustained virological response (SVR) by DAA, and 104 cases were not treated with any antiviral therapy (non-treatment group). Recurrence-free survival (RFS) following curative resection was analyzed by using the log-rank test and Kaplan–Meier method. A Cox proportional hazards model was used to analyze the factors that impacted RFS and OS. *Results*: Five patients (10.4%) experienced HCC recurrence after DAA therapy. The cumulative HCC recurrence rate was significantly lower in the DAA group than the non-treatment group (*p* < 0.001). Multivariate analysis revealed a significant difference in RFS between the non-treatment group and DAA group (*p* = 0.001; hazard ratio (HR), 4.978; 95% CI, 1.976–12.542); liver cirrhosis (*p* = 0.005; HR, 2.062; 95% CI, 1.247–3.410), microvascular invasion (*p* = 0.001; HR, 2.331; 95% CI, 1.408–3.860) and AFP > 15 ng/mL (*p* = 0.022; HR, 1.799; 95% CI, 1.089–2.970) were also independent factors for HCC recurrence. ALBI stage II/III (*p* = 0.005; HR, 3.249; 95% CI, 1.418–7.443) and microvascular invasion (*p* < 0.001; HR, 4.037 95% CI, 2.071–7.869) were independent factors for OS; no significant difference in OS was observed between the DAA and no DAA treatment groups. *Conclusions*: DAA treatment could reduce the risk of recurrence after curative treatment for early stage HCC.

## 1. Introduction

Hepatocellular carcinoma (HCC) is associated with high rates of mortality worldwide and is more common among males than females [1]. Cirrhosis due to chronic infection with hepatitis C virus (HCV) is a leading risk factor for this tumor type. Patients diagnosed with early stage HCC are suitable for curative treatments, such as surgical resection or local ablative therapies; nevertheless, these therapies are restricted by high rates of recurrence (50–60%) [2,3,4,5,6]. The tumor size, serum alpha-fetoprotein, presence of cirrhosis, microvascular invasion and administration of antiviral therapies have been identified as prognostic factors for recurrence after resection [7]. Therefore, suppression of HCV is important, and HCV treatment should be actively introduced.

Before the first generation of direct-acting antivirals (DAAs) was developed, interferon (IFN)-based regimens were the standard of treatment for HCV and led to a sustained virological response (SVR) rate of approximately 50–60% among treated patients [8]. Achievement of a SVR is associated with a reduced risk of HCC recurrence after curative therapy, as well as need for liver transplantation, a lower risk of hepatic decompensation, and both liver-related and overall mortality [9,10,11,12,13]. However, IFN is not suitable for older or liver-cancer patients with a low platelet count, because of the risk of adverse events, such as high fever, general malaise, depression, interstitial pneumonia and decreased platelet count [14]. The recent introduction of DAAs has dramatically improved the SVR rates to over 95% in treated patients, even among traditionally high-risk patients [15,16,17]. The achievement of these high SVR rates has raised hope that there will be a significant reduction in the incidence of HCC. However, it remains unclear whether DAA therapies have a similar benefit in terms of decreasing recurrence in patients with HCC who achieve a SVR after DAA treatment. A number of studies have indicated that IFN-free DAA treatment reduces the risk of HCC recurrence; however, different studies reached opposing conclusions [18,19,20]. This discrepancy has not yet been resolved, and randomized controlled trials (RCTs) with homogeneous follow-up strategies are required to address these limitations. However, RCTs that directly compare groups treated and groups untreated with DAAs are considered unethical and unfeasible.

Because of the low feasibility of carrying out RCTs to address this issue, we conducted a longitudinal study to retrospectively compare the rates of HCC recurrence between patients treated with and without DAA therapy after curative surgical resection in early stage HCC.

## 2. Materials and Methods

### 2.1. Patients

**Figure 1:**
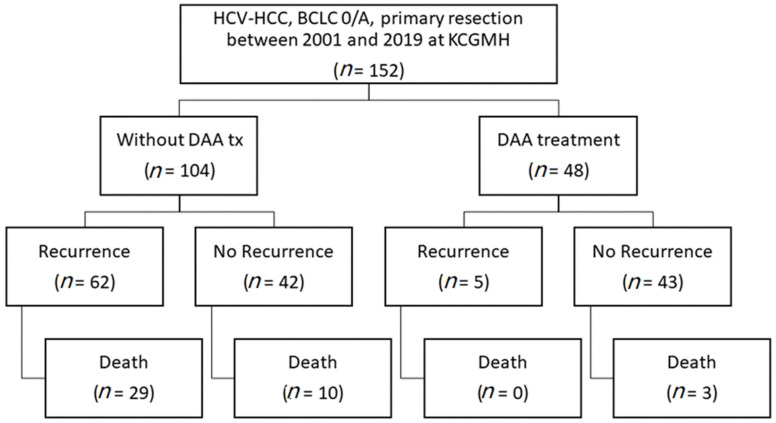
Patient-selection flow diagram.

We conducted a retrospective cohort study of mono-infected patients with HCV-related early stage HCC (BCLC stage 0/A) who underwent curative surgical resection at Kaohsiung Chang Gung Memorial Hospital between May 2001 and October 2019. A total of 152 treatment-naïve patients with HCV-related early stage HCC (BCLC stage 0/A) who underwent curative surgical resection achieved a complete tumor response and who were not treated with any antiviral drugs after curative resection were recruited into this study; 48 patients with HCV-related HCC who received DAA and achieved an SVR were enrolled in the DAA group, while the other 104 patients were enrolled as the untreated group. Figure 1 presents a patient-selection flow diagram.

Follow-up ended on 30 September 2020. Ethics approval for this analysis was granted by the Institutional Review Board of Kaohsiung Chang Gung Memorial Hospital (IRB number: 201901103B0). In addition, this analysis was performed in compliance with the Declaration of Helsinki.

### 2.2. HCC Diagnosis and Follow-Up

All patients were diagnosed with HCC based on magnetic resonance imaging (MRI), dynamic-computed tomography (CT) imaging or pathology, following the guidelines for HCC management. Tumor stage was assessed based on the Barcelona Clinic Liver Cancer (BCLC) staging system. Dynamic CT or MRI was performed to confirm the absence of viable HCC before antiviral therapies were initiated in the DAA group, or after curative therapies in the untreated group. Follow-up for all patients included abdominal ultrasonography and serum alpha-fetoprotein (AFP) quantification at 3-month intervals. Suspected cases of HCC recurrence were confirmed by dynamic CT or MRI according to the same criteria applied for HCC diagnosis.

Overall survival (OS) and recurrence-free survival (RFS) were calculated separately for the two groups: RFS and OS for the DAA group represent the intervals from achieving a SVR until HCC recurrence or death from HCC (or last follow-up visit), while RFS and OS in the untreated group represent the intervals from obtaining a complete tumor response after surgical resection until HCC recurrence or death from HCC (or last follow-up visit).

### 2.3. Antiviral Therapy

All antiviral regimens followed the HCV management guidelines (APASL and EASL). The reimbursement criteria of DAA regimens and genotypes of the National Health Insurance (NHI) were as follows: daclatasvir plus asunaprevir for 24 weeks for genotype 1b without resistance, ombitasvir and paritaprevir with ritonavir for 12 weeks for genotype 1, and elbasvir plus grazoprevir for 12 weeks for genotype 1 or 4. When ledipasvir/sofosbuvir was used for patients with HCV with genotype 2, the treatment duration was 12 weeks, and weight-based ribavirin (Robatrol, 200 mg capsule, Genovate Biotechnology Co. Ltd., Hsinchu, Taiwan) was added for patients with liver decompensation or previous peginterferon plus ribavirin therapy. The DAA was administered pre-operative, immediately post-operative, after several weeks in the study. The definition of SVR12 was that after the cessation of antiviral therapy, a serum HCV-RNA titer below the sensitivity of detection at 12 weeks.

### 2.4. Statistical Analysis

We compared categorical variables by using Chi-squared or Fisher exact tests and continuous variables, with Student’s *t*-tests, Mann–Whitney *U* tests or Kruskal–Wallis tests, as appropriate. The cumulative incidences of HCC recurrence and death were reckoned according to the Kaplan–Meier method, and differences between groups were estimated with the log-rank test. The baseline predictors of HCC recurrence and overall survival were assessed by using univariate and multivariable Cox proportional hazards regression analyses. The *p*-values < 0.05 were considered statistically significant. All statistical analyses were conducted with SPSS 20.

## 3. Results

### 3.1. Characteristics of the Patients

The characteristics of the patients are presented in Table 1. The cohort included 92 men and 60 women, with a median age of 66 years at enrollment. Of the 152 patients, 48 (31.6%) received DAA treatment (DAA treatment group) and 104 (68.4%) did not receive any treatment for HCV (no DAA treatment group). The mean follow-up time in the no-DAA-treatment group was 62.8 months, compared to 19.6 months in the DAA group. In the DAA-treatment cohort, the median time from surgical resection to antiviral therapy was 41.2 months (IQR: 18.4 to 73.4 months). All of the DAA therapies started after surgery, and more than half started after 2 years of surgery (Table 1).

Overall, the rates of recurrence and death were significantly different between the DAA-treatment group and the no-HCV-treatment group (both *p* < 0.001). However, there was no significant difference between the DAA-treatment group and no-HCV-treatment group in terms of any baseline characteristic, including diabetes mellitus, platelets, AST, ALT, total bilirubin, albumin, creatinine, AFP, Child–Pugh A/B, liver cirrhosis, number of tumors, maximum tumor size, histological grade and microvascular invasion.

### 3.2. Subgroup Analysis

**Figure 2:**
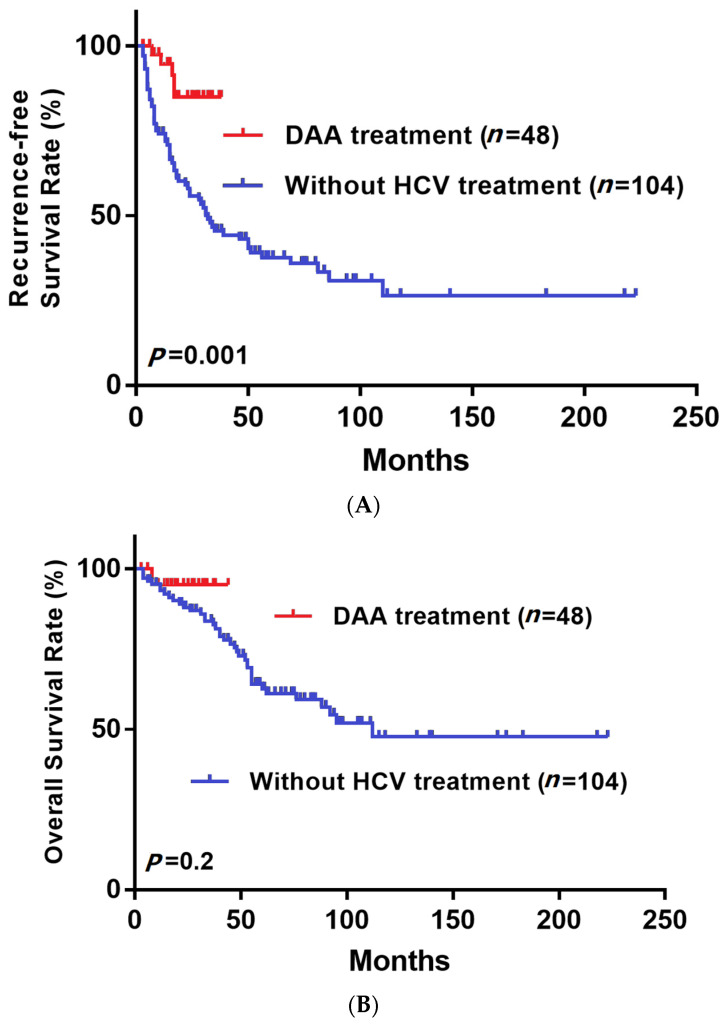
(**A**) Recurrence-free survival (RFS) and (**B**) overall survival (OS) after curative resection for patients with HCC treated with or without DAA.

**Figure 3:**
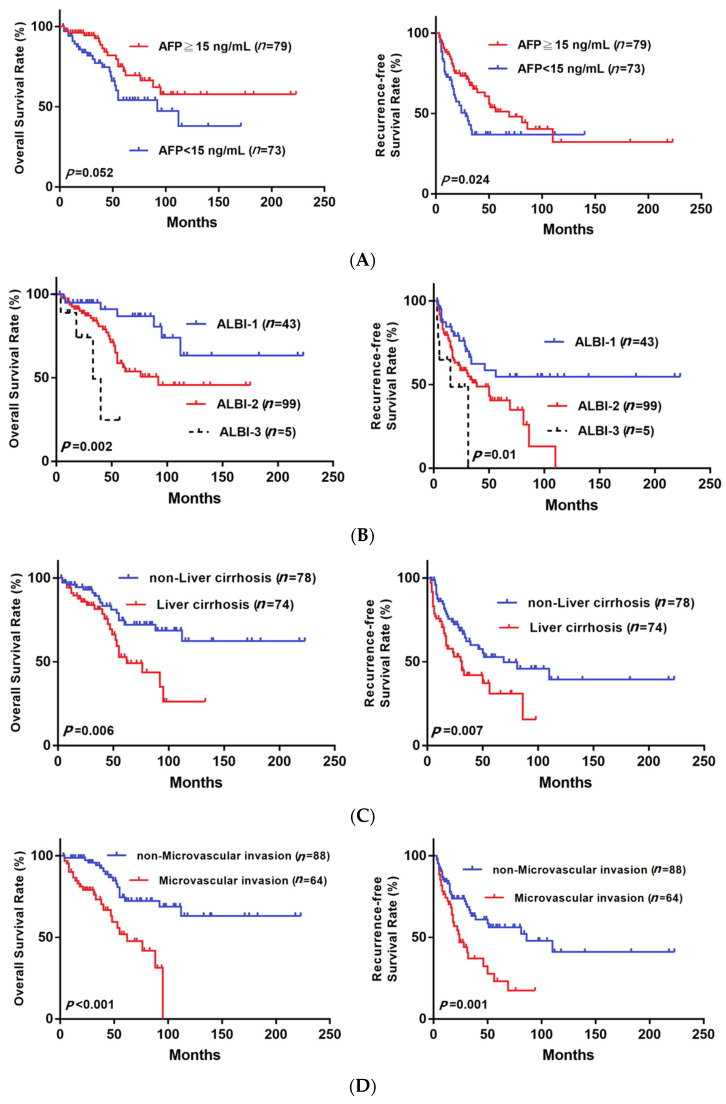
OS and RFS for all cases of BCLC 0/A stage HCC after curative resection stratified by AFP (**A**), ALBI stage (**B**), liver cirrhosis (**C**) and microvascular invasion (**D**).

**Figure 4:**
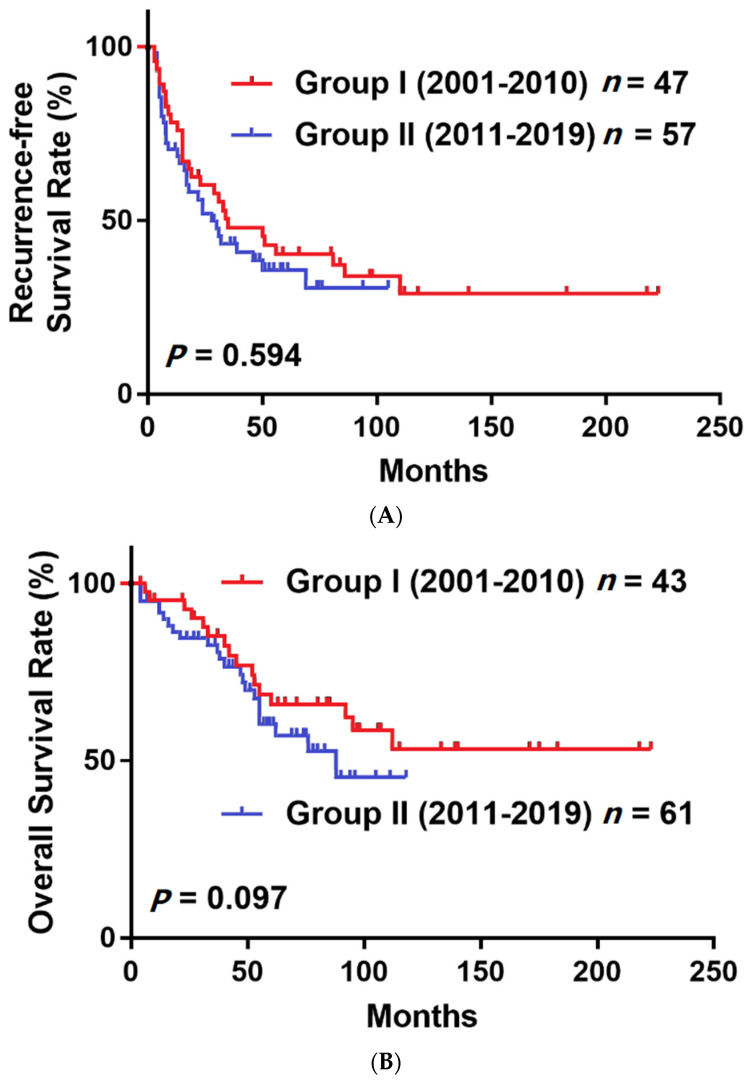
Analysis of (**A**) RFS and (**B**) OS in HCC patients without DAA treatment between 2001 and 2010 and between 2011 and 2019.

Figure 2 shows that the RFS was significantly higher in the DAA group, but the OS was not significantly different between groups. The RFS at 1 year and 2 years after surgery in the non-DAA group was 73.9% and 57.8%, and in the DAA group, it was 94.6% and 84.4%, respectively. The OS at 1 year and 2 years after surgery in the non-DAA group was 93.1% and 87.9%, and in DAA group, it was 92.9% and 92.9%, respectively. In our subgroup analysis based on various clinical characteristics (Figure 3), the RFS was significantly higher in the subgroups of patients with AFP > 15 (*p* = 0.024, Figure 3A), ALBI stage II/III (*p* = 0.01, Figure 3B), liver cirrhosis (*p* = 0.007, Figure 3C) and microvascular invasion (*p* = 0.001, Figure 3D). Moreover, the OS was significantly higher in the ALBI stage II/III (*p* = 0.002, Figure 3B), liver cirrhosis (*p* = 0.006, Figure 3C) and microvascular invasion (*p* < 0.001, Figure 3D) subgroups. To address the selection bias caused by different timelines (from 2001 to 2019), we divided patients who did not receive antiviral treatment into two groups by time of surgery, 2001–2010 and 2011–2019. The Figure 4A showed that there was no significant difference in RFS (*p* = 0.594) between group one (2001–2010) and group two (2011–2019), and the Figure 4B demonstrated that there was no significant difference in OS (*p* = 0.097) between group one (2001–2010) and group two (2011–2019). This result indicated that the difference of timeline and surgical techniques did not affect the surgical outcomes.

### 3.3. Univariate and Multivariate Analyses of Independent Risk Factors

Univariate analyses demonstrated significant associations between serum AFP, liver cirrhosis, ALBI stage, tumor size, microvascular invasion and DAA treatment with RFS (Table 2). In the multivariate analyses, serum AFP > 15 ng/mL (HR, 1.799; 95% CI, 1.089–2.970; *p* = 0.022), liver cirrhosis (HR, 2.062; 95% CI, 1.247–3.410; *p* = 0.005), microvascular invasion (HR, 2.331; 95% CI, 1.408–3.860; *p* = 0.001) and no-DAA treatment (HR, 4.978; 95% CI, 1.976–12.542; *p* = 0.001) remained independent prognostic factors for RFS.

In terms of OS, the multivariate Cox proportional hazards model revealed that ALBI stage II/III (HR, 3.249; 95% CI, 1.418–7.443; *p* = 0.005) and microvascular invasion (HR, 4.307; 95% CI, 2.071–7.869; *p* < 0.001) were independent risk factors associated with OS (Table 3). However, there was no significant association between DAA treatment and OS.

## 4. Discussion

In this present real-world observational study, we looked into the consequents of DAA therapy on the prognosis and rate of recurrence among patients with early stage HCC after curative resection. To date, the criteria for DAA use in patients with HCC are based on the recommendations for the general CHC population, who were seropositive of anti-HCV and HCV. However, even though DAAs has introduced high SVR rates, even for patients with HCC, there was no consensus of DAA therapy for HCV viremia patients with treated or inactive HCC; concerns have especially been expressed regarding the HCC recurrence risk following DAA therapy in 2016. Hence, not all HCC patients received DAA therapy after curative resection. In this retrospective study, we only recruited HCC patients with SVR by DAA treatment with different regimens, aiming to confirm whether higher HCC recurrent rate after DAA treatments occurred compared to those without DAA treatment. Our results indicate that DAA could lead to a lower recurrence risk in early stage hepatitis C virus-related HCC after curative resection.

Improved understanding of the hepatitis C virus (HCV) genome and proteins has enabled efforts to improve the efficacy and tolerability of treatment for HCV. Therefore, the WHO expect that HCV could be eradicated by 2030. DAAs lead to higher SVR rates than IFN-based regimens, and achieving an SVR has been reported to lead to a reduction in HCC risk. Unfortunately, the precise effects of DAA therapy on early recurrence and OS in HCC are controversial. The conflicting results obtained in different studies are partly related to differences in tumor stage, microvascular invasion, HCC treatment modalities, the interval between HCC treatment and initiation of DAA, and indirect comparisons of the DAA and control groups [21]. A meta-analysis conducted in 2017 concluded that there was no evidence that DAA and IFN-based therapy lead to differential rates of HCC occurrence or recurrence following achievement of an SVR [22]. Another meta-analysis—which stratified the studies according to study- and patient-level variables identified in meta-regression analyses to describe the wide variability in the natural course of HCV-related early HCC after curative treatment—indicated that mean AFP level and tumor size were significant predictors of survival and confirmed that survival in patients with compensated cirrhosis was significantly impacted by cancer-related factors. Moreover, serum albumin was found to be an autonomous indicator of HCC recurrence in patients with HCV- or HBV-related HCC after endeavored therapeutic medicines, though the follow-up length and study configuration remained essentially connected with the recurrence rate [23]. Therefore, to reduce the influence of cofounders caused by HCC tumor effects, the present study focused on early stage HCC (BCLC stage 0/A) among patients who underwent curative resection. Indeed, the baseline characteristic was not significantly different between the DAA group and no DAA group; thus, our study provides a more reliable assessment of whether DAA reduces the rate of recurrence in HCC.

Five cases of HCC recurrence were observed after a mean follow-up of 20 months in this study, indicating that extirpation of HCV with DAA does not rule out the risk of developing HCC recurrence after effective therapy. One possible hypothesis is that clearance of HCV could dampen the immune system response, which may induce the progression or development of HCC, especially in individuals with neoplastic foci [24]. Giovannini et al. reported that DAA therapy can modulate proliferation, invasive ability and gene expression in HCC in vitro; thus, the off-target effects of DAAs may both stimulate and inhibit tumor cell migration and proliferation. Furthermore, the same article also mentioned that off-target gene modulation by DAA—which mainly affects histones, mitochondrial function and ribosomal genes—induced phenotypic changes that may underlie both the tumor-suppressive and pro-oncogenic activities of DAAs [25]. These factors could also explain why patients with HCV-related HCC who achieve a SVR after treatment with DAAs still have a risk of HCC recurrence. Hence, radiological and active clinical follow-up of HCC recurrence is still necessary, regardless of whether HCV is eradicated by DAAs or not.

A retrospective study in Japan compared DAA treatment and IFN treatment after curative treatment and showed that DAA therapy decreased the risk of HCC recurrence and improved OS. However, despite employing propensity score matching, that analysis could be cofounded by differences in the SVR rate between the DAA treatment group and IFN treatment group [26]. Another multicenter study declared that DAA therapy after curative treatment for early HCC ameliorated OS compared to patients who did not obtain DAAs, as DAA treatment was found to lower the risk of decompensation events in patients with advanced liver disease and HCC [27]. Nevertheless, the patients in that study all had HCV-related cirrhosis. In this study, there was no significant difference in OS between the no-DAA group and the DAA group, due to the short-term follow-up. A recent study by Hironori Ochi et al. reported that DAAs improved the survival and recurrence rates and maintained hepatic functional reserve in patients with early stage HCC over a mid- to long-term observation period. Nevertheless, pathological examinations were not conducted for the patients who underwent RFA treatment in that study; thus, risk factors, such as tumor differentiation and vascular invasion, were not investigated [28].

SVR is associated with improved OS and RFS in patients with HCV who have undergone resection or locoregional therapy for HCC [29]. Moreover, the response to DAA therapy is lower among patients with HCC compared to those without HCC, regardless of cirrhosis status [30]. Therefore, in our study, we enrolled patients with HCV-HCC who received DAAs and achieved a SVR after curative treatment for HCC in the DAA group, and our multivariate analyses showed that RFS was significantly lower in the DAA group. Furthermore, a previous cohort study indicated that diabetes was independently associated with HCC recurrence after DAA treatment [31]. However, there were no significant differences in any baseline characteristic, including diabetes, between the no-HCV-treatment group and the DAA group, thus reducing the influence of potential cofounders. In addition to DAA treatment, our subgroup analysis also found that liver cirrhosis, serum AFP, ALBI grade and microvascular invasion represented independent risk factors for recurrence in HCV-related HCC. A review by Kishta et al. indicated that HBC infection, HCV infection, NAFLD, alcoholic liver disease, cirrhosis, exposure to aflatoxins and continuous chronic alcohol abuse (consumption of 40–60 g of alcohol daily) were risk factors that are associated with the recurrence of HCV-HCC. Moreover, the review also demonstrated that long-term HCV infection allows genetic and/or epigenetic changes to accumulate in hepatic tissues, suggesting that chronically damaged liver tissues have substantial precancerous potential. Moreover, a study conducted in Korea showed that short-lasting HCC treatment (<12 months) was associated with HCC recurrence after DAA therapy [32,33].

There are some limitations to this study. First, this was a retrospective study of patients from a single institution and the data were collected from medical records. Despite the use of multivariable analysis, not all confounding factors could be completely adjusted for. In addition, a propensity-score-matching analysis was not carried out in the present study, due to no significant differences in terms of the baseline characteristics (shown in the Table 1); moreover, the case number was too small to conduct a PSM analysis. Enrollment of a larger number of patients in the DAA group is necessary in future studies. Secondly, this was a single-center study; as there was no universal consensus on DAA treatment after HCC resection during the period in which the patients in this study were treated and no option for DAA before 2014, selection bias may potentially exist. A validation cohort is necessary to confirm our major findings. Finally, the mean follow-up time for the DAA group was too short to confirm the efficacy of DAA treatment on OS. Further clinical trials may substantiate our findings and prove the benefit of DAA therapy on long-term outcomes.

## 5. Conclusions

DAA treatment after curative treatment for early stage HCC could reduce the risk of HCC recurrence. However, a risk of recurrence still exists, indicating that active clinical follow-up remains clinically significant. Further multicenter studies with larger numbers of patients and longer follow-up durations are necessary to identify patients who are at a high risk of HCC recurrence.

## Figures and Tables

**Table 1:** Characteristics of the study population.

	No HCV Tx (*n* = 104)	DAA Tx (*n* = 48)	*p*-Value
Age (years)	66.1 ± 808	65.2 ± 8.7	0.561
Sex, male	66 (63.5%)	26 (54.2%)	0.276
Diabetes mellitus	35 (33.7%)	13 (27.1%)	0.418
Platelet (1000/μL)	144.5 ± 57.7	129.8 ± 42.7	0.118
AST (U/L)	63.1 ± 55.3	66.8 ± 50.8	0.695
ALT (U/L)	61.1 ± 46.5	70.5 ± 51.8	0.270
Total bilirubin (mg/dL)	0.8 ± 0.3	1.2 ± 2.8	0.282
Albumin (g/dL)	3.5 ± 0.7	3.5 ± 06	0.814
Creatinine (mg/dL)	1.1 ± 1.2	1.2 ± 1.7	0.953
AFP (>200 ng/mL)	22 (22.4%)	13 (27.1%)	0.538
Child–Pugh A/B	87/17	41/7	0.782
Liver cirrhosis	48 (46.2%)	26 (54.2%)	0.358
DAA regimen			
ASV + DCV		11 (22.9%)	
SOF + RBV		7 (14.6%)	
SOF + LDV		14 (29.2%)	
SOF + VEL		5 (10.4%)	
ERB + GZR		8 (16.7%)	
GLE + PIB		3 (6.3%)	
DAA-surgery interval, months			
DAA before surgery		0	
DAA after surgery		48 (100%)	
0–12 months after surgery		10 (20.8%)	
12–24 months after surgery		5 (10.4%)	
>24 months after surgery		33 (68.8%)	
Tumors, multiple	6 (4.8%)	5 (10.4%)	0.195
Tumor size max (cm)	2.9 ± 0.9	2.8 ± 1.1	0.485
Histology grade (I/II/III)	14/87/3	4/43/1	0.624
Microvascular invasion	40 (38.5%)	24 (50%)	0.180
Recurrence	62 (59.6%)	5 (10.4%)	<0.001
Death	39 (37.5%)	3 (6.3%)	<0.001
Follow-up (months)	62.8 ± 44.9	19.6 ± 11.3	<0.001

Data are expressed as mean ± standard deviation or *n* (%). Abbreviations: HCV Tx, hepatitis C virus treatment; DAA Tx, direct-acting antiviral treatment; AFP, alpha fetoprotein; AST, aspartate aminotransferase; ALT, alanine aminotransferase; ASV, asunaprevir; DCV, daclatasvir; SOF, sofosbuvir; RBV, ribavirin; LDV, ledipasvir; VEL, velpatasvir; ERB, elbasvir; GZR, grazoprevir; GLE, glecaprevir; PIB, pibrentasvir.

**Table 2:** Uni- and multivariate analyses of prognostic factors for recurrence in HCC.

		Univariate		Multivariate	
		HR (95%CI)	*p*	HR (95%CI)	*p*
Age (years)	>60 vs. ≤60	1.093 (0.645–1.852)	0.742		
Gender	Male vs. Female	1.063 (0.644–1.755)	0.813		
DM	Yes vs. No	0.822 (0.478–1.414)	0.479		
Platelet (1000/μL)	<150 vs. ≥150	1.435 (0.854–2.413)	0.173		
AFP (ng/mL)	>15 vs. ≤15	1.764 (1.083–2.872)	0.023	1.799 (1.089–2.970)	0.022
Albumin (g/dL)	<3 vs. ≥3	1.708 (1.014–2.876)	0.044		
Child–Pugh	B vs. A	0.951 (0.471–1.921)	0.889		
ALBI stage	II/III vs. I	2.086 (1.147–3.796)	0.016		
BCLC stage	A vs. 0	1.704 (0.813–3.570)	0.158		
Tumor size (cm)	>2 vs. ≤2	1.715 (0.933–3.151)	0.082		
Tumor number	Multiple vs. Single	1.375 (0.551–3.430)	0.495		
Liver cirrhosis	Yes vs. No	1.954 (1.194–3.197)	0.008	2.062 (1.247–3.410)	0.005
Histology grade	Moderate + Poor vs. Well	1.577 (0.711–3.494)	0.262		
Microvascular invasion	Present vs. Absent	2.246 (1.370–3.683)	0.001	2.331 (1.408–3.860)	0.001
DAA treatment	No vs. Yes	4.058 (1.615–10.199)	0.003	4.978 (1.976–12.542)	0.001

Abbreviations: HR, hazard ratio; CI, confidence interval; DM, diabetes mellitus; DAA, direct-acting antivirals; AFP, alpha fetoprotein.

**Table 3:** Uni- and multivariate analyses of prognostic factors for all-cause mortality.

		Univariate		Multivariate	
		HR (95% CI)	*p*	HR (95%CI)	*P*
Age (years)	>60 vs. ≤60	1.670 (0.798–3.494)	0.174		
Gender	Male vs. Female	1.642 (0.839–3.214)	0.148		
DM	Yes vs. No	1.883 (1.019–3.479)	0.043		
Platelet (1000/μL)	<150 vs. ≤150	1.718 (0.895–3.297)	0.104		
AFP (ng/mL)	>15 vs. ≤15	1.909 (1.034–3.525)	0.039		
Albumin (g/dL)	<3 vs. ≥3	1.985 (1.045–3.772)	0.036		
Child–Pugh	B vs. A	1.571 (0.724–3.410)	0.253		
ALBI stage	II/III vs. I	2.876 (1.262–6.553)	0.012	3.249 (1.418–7.443)	0.005
BCLC stage	A vs. 0	1.322 (0.556–3.143)	0.527		
Tumor size (cm)	>2 vs. ≤2	1.428 (0.681–2.992)	0.345		
Tumor number	Multiple vs. Single	1.375 (0.551–3.430)	0.495		
Liver cirrhosis	Yes vs. No	2.219 (1.187–4.149)	0.013		
Histology grade	Moderate + Poor vs. Well	1.813 (1.641–5.124)	0.262		
Microvascular invasion	Present vs. Absent	3.606 (1.890–6.879)	<0.001	4.037 (2.071–7.869)	<0.001
DAA treatment	No vs. Yes	1.714 (0.496–5.925)	0.394		

Abbreviations: HR, hazard ratio; CI, confidence interval; DM, diabetes mellitus; DAA, direct-acting antivirals; AFP, alpha fetoprotein.

## Data Availability

The original data for this manuscript can be obtained upon reasonable request to the corresponding author.
